# Deuterium-Depleted Water Influence on the Isotope ^2^H/^1^H Regulation in Body and Individual Adaptation

**DOI:** 10.3390/nu11081903

**Published:** 2019-08-15

**Authors:** Alexander Basov, Liliia Fedulova, Mikhail Baryshev, Stepan Dzhimak

**Affiliations:** 1 Kuban State Medical University, 350063 Krasnodar, Russia; 2 Kuban State University, 350040 Krasnodar, Russia; 3 The V.M. Gorbatov Federal Research Center for Food Systems of Russian Academy of Sciences, 109316 Moscow, Russia; 4 Federal Research Center the Southern Scientific Center of the Russian Academy of Sciences, 344006 Rostov-on-Don, Russia

**Keywords:** deuterium, water, adaptation, DNA

## Abstract

This review article presents data about the influence of deuterium-depleted water (DDW) on biological systems. It is known that the isotope abundances of natural and bottled waters are variable worldwide. That is why different drinking rations lead to changes of stable isotopes content in body water fluxes in human and animal organisms. Also, intracellular water isotope ratios in living systems depends on metabolic activity and food consumption. We found the ^2^H/^1^H gradient in human fluids (δ^2^H _saliva_ >> δ^2^H _blood plasma_ > δ^2^H_breast milk_), which decreases significantly during DDW intake. Moreover, DDW induces several important biological effects in organism (antioxidant, metabolic detoxification, anticancer, rejuvenation, behavior, etc.). Changing the isotope ^2^H/^1^H gradient from “^2^H _blood plasma_ > δ^2^H _visceral organs_” to “δ^2^H _blood plasma_ << δ^2^H _visceral organs_” via DDW drinking increases individual adaptation by isotopic shock. The other possible mechanisms of long-term adaptation is DDW influence on the growth rate of cells, enzyme activity and cellular energetics (e.g., stimulation of the mitochondrion activity). In addition, DDW reduces the number of single-stranded DNA breaks and modifies the miRNA profile.

## 1. Introduction

Non-radioactive isotopes of biogenic elements (^2^H/^1^H, ^18^O/^17^O/^16^O, ^13^С/^12^С, ^15^N/^14^N) have a significant influence on the rate of biochemical reactions, physiological processes, growth and development of unicellular and multicellular living organisms with different levels of energy metabolism and metabolic intensity rate [1,2,3,4].

Hydrogen and oxygen isotopes occupy a special place among all non-radioactive nutrients which are primarily included in addition to organic and inorganic compounds in the composition of water, as water is an essential solvent for all biological objects where the vast majority of biochemical reactions occur. In this regard, the importance of water for the implementation of physiological processes is extremely high, but it might vary and differ for each of its nine isotopologues: ^1^H_2_^16^O, ^1^HD^16^O, D_2_^16^O, ^1^H_2_^17^O, ^1^HD^17^O, D_2_^17^O, ^1^H_2_^18^O, ^1^HD^18^O, D_2_^18^O. Some of them, which predominantly contain lighter isotopes, can have in certain concentrations a stimulating effect on functional activity of living systems [5], other isotopologues, including mainly heavy isotopes, are able to inhibit vital processes, especially at high concentrations of deuterium [6,7], and in some cases it was observed that variation of deuterium concentration (both increasing and decreasing its content in water) can enhance the functional activity of living systems [8]. In this regard it is very important to know about the peculiar features of use of certain isotopologues of water for regulation of metabolic processes in a body, and for monitoring of the anabolism and catabolism state of biological substances in various diseases [9].

The ratios of deuterium and protium in natural and bottled waters can vary by up to about two times, which is important to consider when people and animals migrate between continents and climatic zones. For example, the minimum deuterium content in natural water is observed in the ice of Antarctica: Standard Light Antarctic Precipitation (SLAP), which is ^2^H_SLAP_/^1^H_SLAP_ = 89.0⋅10^−6^ or 89 ppm, or −428.5‰ [10,11]; a somewhat higher content of deuterium (by 40%) is registered in Greenland ice and corresponds to the standard Greenland Ice Sheet Precipitation (GISP): ^2^H_GISP_/^1^H_GISP_ = 124.6⋅10^−6^ (124.6 ppm or −189.5‰ [12]); whereas the main standard for the content of deuterium in natural water, the Vienna Standard Mean Ocean Water (VSMOW), is: ^2^H_VSMOW_/^1^H_VSMOW_ = 155.76⋅10^−6^ (155.76 ppm or 0.0‰ [13]), which exceeds the values of ^2^H_SLAP_ by 75%. It is necessary to note that the highest content of the natural deuterium (in Dababa +16‰) was found when studying the groundwater of the Sahel-Sahara region during the drought period [14,15,16].

Fluctuations of ^18^О content are significantly lower than fluctuations of hydrogen isotopes content, and are equal to: ^18^O_SLAP_/^16^O_SLAP_ = 1894⋅10^−6^ (1894 ppm or −55.36‰ [16]); ^18^O_GISP_/^16^O_GISP_ = 1955.4⋅10^−6^ (1955.4 ppm or −24.76‰ [12]), which is only by 3% higher than standard ^18^O_SLAP_; ^18^O_VSMOW_/^16^O_VSMOW_ = 2005⋅10^−6^ (2005 ppm or 0.0‰), at this value exceeds the value ^18^O_SLAP_ by 5.9%, thus being significantly lower (by 12.7 times) than the differences in the ratio of similar standards ^2^H and ^1^H.

In the natural water sources some peculiar features were observed for a range of closed and freshwater water sources which feature significant differences from VSMOW as well as seasonal variations in isotopic composition of water. For example, in the surface waters of Kyambangunguru lake which is the part of Mbaka lakes, at the end of the dry season deuterium content was 16‰, oxygen-18 content was 3.4‰, while at the end of the rainy season the content of δ^2^H was −7‰, content of δ^18^O was equal to −0.8‰ [17]. Stable isotopic composition of water from the Antarctic subglacial Lake Vostok was: −59.0‰ (by 6% less ^18^O _VSMOW_) for oxygen-18 and −455‰ (by 45% less ^2^H _VSMOW_) for deuterium [18]. Isotopic composition of Lake Baikal, Siberia, varies only a little during a year, and is equal to: δ^18^O = −15.8‰, δ^2^H = −123‰, which is 1.6% and 12.3% less, than ^18^O_VSMOW_ and ^2^H_VSMOW_ respectively [19]. The variations of the isotopic composition in natural water sources are explained by displacement of isotopic equilibrium in range of cases as a result of phase transitions in the water cycle, which leads to fractionation in nature, primarily of ^2^H/^1^H and ^18^O/^17^O/^16^O [20,21]. All this leads to a two-stage decrease in content of heavy isotopologues in water vapor: first due to a decrease in their concentration in the water evaporating from the surface of the seas and oceans, and then during partial condensation when precipitation forms (most expressed at their first fall, which also depends on terrain features, geographical and climatic conditions, precipitation frequency, etc. [22]). In this regard snow and rain falling far from the place of their evaporation are characterized by a high content of light water molecules (^1^H_2_^16^O). Furthermore, the flora consumes this water, however as a result of transpiration it gradually enriches this water with ^2^Н and ^18^О, leading to introduction of these isotopes into organic compounds [23,24]. This, for instance, explains the significant difference in isotopic composition of juices freshly squeezed from fruits and vegetables grown in various geographic regions [25,26,27]. In its turn water in healthy animal tissues is enriched with isotopes from two main sources: ingested/drinking water and water formed during oxidation of reducting equivalents during the metabolism of food substrates, including the reduced form of nicotinamide adenine dinucleotide (NAD) in the mitochondrial respiratory chain [28,29,30,31]. Moreover, the share of intracellular water synthesized de novo can reach more than 50% [32,33], that occurs with the active interaction of mitochondria, endoplasmic reticulum and peroxisome [34]. In the last organelle production of metabolic water (for example: its catalase converts H_2_O_2_-excess into H_2_O и molecular oxygen) increases under some pathological and specific conditions, such as high-fat diet (especially including very-long-chain and methyl-branched fatty acids), administration of hypolipidemic drugs (like clofibrate), ingestion of xenobiotics, development of inflammation, and others. Peroxisomal oxidase H_2_O_2_-production achieves about 1/3 of all cellular hydrogen peroxide [35], and oxygen consumption in peroxisomes can be about 1/5 of all O_2_ in cells of metabolic active tissues. Additional pathways of isotopic fractionation in the animal world are perspiration and respiration processes [29], for example due to the release of carbon dioxide, which is highly enriched by ^18^О [36]. The release of carbon dioxide is caused by the fact than in a body there is an exchange of oxygen atom between СО_2_ and H_2_O with participation of carbonic anhydrase, while fractionation of ^18^O depends on time of interaction and pressure of carbon dioxide [37,38]. It is necessary to note that the carbonyl and carboxyl groups of organic substrates are the most active in oxygen atoms exchanging with intracellular water, whereas hydroxyl groups do not participate in oxygen-18 fractionation processes under physiological conditions [39]. At the same time the exchange of protons and deuterons is much more active in live organisms, since functional groups −OH, −NH_3_^+^, =NH, −SH easily dissociate, affecting the isotopic composition of the liquid medium [40], and although the carbon-related hydrogen which is most commonly found in live objects is stable (with a small exception of hydrogen atoms in composition of CH_2_-groups adjacent to carbonyl groups), it is also able to indirectly affect the isotopic composition of water during the oxidation of organic substrates [1,41].

It should be pointed out that fluctuations in isotopic composition of water in a human body can have a wider range, both in connection with the consumption of not only water from surface water sources [42], but from water obtained from any other origin (artesian, glacial, mineral springs), and due to the characteristics of the food ration, for example the predominant consumption of lipid-based nutrients [43]. During study of isotopic composition of samples of bottled and packaged waters of the world, several scientific groups defined the following range of fluctuations: δ^2^H from −147‰ to 15‰ and δ^18^O from −19.1‰ to 3.0‰ [44,45,46]. Although as it was shown earlier the bottled mineral waters usually have insignificant differences in deuterium content and even smaller fluctuations in concentration of oxygen-18, modern technologies allow us to obtain deuterium-depleted water (DDW) (with deuterium content till δ^2^H = −968‰) and oxygen-18 depleted water (reduction till 26.1% in comparison with ^18^O_VSMOW_) [47,48,49,50,51].

The use of this water can have a significant impact on the isotopic composition and functional activity of both a healthy organism and in various diseases [43,52].

## 2. Biological Effects of Deuterium-Depleted Water (DDW)

To date a great number of works have been published, where both the activating and inhibitory effects of DDW on various levels of the organization of living matter (molecular, organoid, cellular, tissue and organismic) are described. Below the various results of studies of DDW effect on biological objects will be discussed.

### 2.1. Activating Influence of DDW at Molecular and Organoid Levels

In one research there was demonstrated an ability of DDW to activate transcription factors (DAF-16 and SOD-3 (extracellular superoxide dismutase [Cu-Zn]), previously inhibited by the introduction of Mnin a *Caenorhabditis elegans*), which were responsible for expression of antioxidant enzymes (superoxide dismutase) and lifespan *of C. elegans*. It is necessary to note that introduction of DDW with δ^2^H = −422‰ regulated DAF-16 pathway, restored the activity of SOD (at the background of manganese intoxication) and lifespan of worms till the control reference values without changing of DAF-2 levels [53].

In other research on rats it was shown that the use of DDW containing deuterium δ^2^H = −807‰ for 60 days led to the stimulation of the antioxidant protection system in erythrocytes, the stimulation of which was accompanied by an increase in the restored GSH (glutathione) and activity of SOD, while a decrease in catalase activity was found in absence of significant changes in the activity of glutathione peroxidase, glutathione reductase. At the same time less prolonged consumption of DDW by animals (within 30 days) led to prooxidant effects, which were accompanied by stimulation of glutathione reductase and catalase activity, while no changes in SOD activity were observed at the background of increase in malondialdehyde content in blood [54].

The study of the 12-week-old normotensive *Wistar-Kyoto* rats and spontaneously hypertensive rats revealed the ability of DDW with deuterium content δ^2^H = −646‰ to increase insulin levels and to lower down the levels of lipids (triglycerides and cholesterol) in the blood of normotensive Wistar-Kyoto rats. At the same time DDW increased the NO· synthase (nitric oxide synthase) activity in the left ventricle of both normotensive Wistar-Kyoto and spontaneously hypertensive rats, although it reduced inducible isoform of nitric oxide synthase (iNOS) protein expression and activity of synthase NO· in the aorta only in spontaneously hypertensive rats. These effects of the exchange of NO· persisted even against the introduction into the drinking ration of a 15% solution of fructose for 6 weeks. That can be explained by the influence of DDW on the terminal complex of mitochondrial electron transport chain, which reduces molecular oxygen into deuterium-depleted water. That, in turn, can change the rate of fatty acid oxidation and gluconeogenesis in a body, including by modifying the level of signaling molecules (reactive oxygen species, NO· and others) [55]. The similar model considered in one of the research works [56] indicates probability of the fact that under conditions of a liquid medium depleted in deuterium, the mechanism of its biological action may be related to the operation of the tricarboxylic acids cycle, because the enzymes localized inside the mitochondria are involved in the fractionation of deuterium. The violation of mitochondrial function due to cell exposure to hypoxia, acidosis, or other pathogenic factors, may reduce the content of a deuterium-free form of reduced nicotinamide adenine dinucleotide phosphate (NADPH), and consequently reduce transfer of ^1^H (H-transfer carried out in transhydrogenase reaction by NAD(P)^+^ transhydrogenase (EC 1.6.1.1): NADPH + NAD^+^ <=> NADP^+^ + NADH) in NADPH, thus reducing the reserves of the universal reducting equivalent [57], irreplaceable for many anabolic processes, and creating the prerequisites for mutation rate increase along with occurrence of further dysplastic processes or a decrease in the mass of organs and the whole organism. A possible mechanism of these phenomena may be the change in transportation of ^1^H atoms formed in mitochondria during the beta-oxidation of fatty acids with a lower content of deuterium and atoms of ^2^H, concentration of which is higher in water and glucose, entering the cytosol from the extracellular environment, leading in general to an increase in the intracellular ratio ^2^H/^1^H [58,59,60]. The higher values of this ratio can lead to changes in the speed and, rarely, to changes in the direction of biochemical reactions in a body, which can also be characterized by impaired replication processes and repair of DNA, which leads to disruption in synthesis of mRNA, and, consequently, of proteins, creating metabolic prerequisites for reducing of body mass and adaptation. Thus, in cells with mitochondria with impaired transfer of ^1^H atom it is possible to regulate the production of high-energy compounds by reducing the concentration of deuterium in the drinking ration, which eventually corrects cell growth, cell division and functional impairment [61]. As a result, a drop in deuterium concentration in cytoplasmic water contributes, among other things, to the normalization of the phenotype of cells with impaired regulation of metabolism. All this leads, for example, to a decrease in the speed of cancer cells division (up to the speed of normal cells division) [62]. The above assumption can be confirmed by the results of a study of the effect of water with different deuterium content on chemical, biological and physical parameters of biological objects [63] (in the experiment the following waters were used: DDW (δ^2^H = −968‰); water with natural content of deuterium (δ^2^H = −101‰); D_2_O (99.99%)). Biological objects demonstrated the characteristics of reactivity at various levels of the organization of biota. It was found that for biological molecules of different classes in the medium with different concentrations of deuterium there were distinctive features of the response to the isotopic composition of the medium: for nucleic acids it was shown that the mutation rate constant for L-galactose was slowed down in D_2_O, whereas the rate of lysozyme activity of destabilase-lysozyme increased by 2 times in a liquid medium с δ^2^H = −968‰, while in D_2_O no changes in activity of destabilase-lysozyme were found. In a two-phase heterogeneous system the kinetics of dissolution of the active ingredients approximately matched with the expected kinetic isotope effect (k^1^H/k^2^H > 1), which confirms the significant contribution of the isotopic composition of water to the process of organic substances dissolution in this water. However, it was noted that the most expressed ^2^H/^1^H-effect was observed in the cell system, where the speed of transition from the active to the immobilized state in *S. ambigua* cells increased 800 times in a medium with δ^2^H = −968‰ in comparison with a medium in which δ^2^H = −101‰ [63].

Particular attention must be drawn to the possible effect of isotopic composition of the medium on the molecular dynamics of the DNA molecule which under natural conditions has one deuterium atom for every 6400 hydrogen atoms and that ratio can lead to a change in the frequency of DNA molecule mutations during living systems evolution [64]. It has been shown that singular replacements of protium atom by deuterium in hydrogen bonds between pairs of nitrogenous bases of the DNA molecule lead to change in the frequency of occurrence of its open states, which in their turn are an obligatory condition for functional activity for the molecule, including facilitating specific intermolecular DNA protein interactions during transcription, folding and replication [65]. It was found that the probability of occurrence of open states between nitrogenous bases in double-stranded DNA depends on concentration of deuterium in the liquid medium surrounding the molecule, and on the magnitude of the energy of hydrogen bonds rupture. When the energy of hydrogen bonds rupture is equal to 0.335 ∙ 10^−22^ J the almost linear decrease in probability of appearance of open states between nitrogen bases in double-stranded DNA (for the first 10 base pairs of the gene encoding alpha 17 interferon) is observed within the range δ^2^H from −743‰ to 0.0‰ in liquid medium surrounding a molecule. In this case the probability of hydrogen bonds rupture between nitrogenous bases in case of introduction of even one deuterium atom into the DNA molecule exceeds the probability of a similar rupture in the same molecule containing only protium atoms, which indicates a stability decrease in DNA molecular structure. If the energy of hydrogen bonds rupture is equal to 0.345 ∙ 10^−22^ J, within the range δ^2^H from −743‰ till 0.0‰ in the liquid medium surrounding the DNA molecule there is almost a linear increase in the probability of open states occurrence between its nitrogenous bases. However, the probability of breaking hydrogen bonds between nitrogenous bases in the case of a deuterium atom introduction into DNA molecule does not exceed the likelihood of a similar rupture in the same molecule containing only protium atoms [66].

One more research work shows the influence of DDW with δ^2^H = −679‰ on the efficiency of immunocompetent proteins extraction from the immune organs of *Sus scrofa*: spleen, thymus and lymph nodes [67]. At the same time, protein–peptide complexes were fractionated using the methods of step-by-step ultrafiltration and gel filtration method. It was shown that the extraction of proteins from the studied samples in solution with δ^2^H = −679‰ provides a nearly twofold increase in the amount of extractable proteins and peptides, which can be caused by change in the ionic strength of this solution along with variation of ^2^H amount in the medium. The main protein components of the extracts were 8 fractions, which contained from 11% to 20% of the total protein material, at that was visually detectable on two-dimensional electrophoresis [68]. The extracted proteins were the members of the family α- and β-chains of hemoglobin, and only in one case the presence of profilin 1 (actin-binding protein) was detected. Other fractions of hemoglobins were post-translationally modified. In a fraction of hemoglobin β-chains we observed the deimination of amino acid residues (Q) of glutamine or (N) asparagine, and their combination was varied in various fractions. Thus the obtained results indicate that the use of fragmented cells of pig immunocompetent organs isolated in the medium with modified ^2^H/^1^H composition as a base matrix allows creating innovative immunostimulating active complexes by improving the quality and safety of livestock products [69].

In addition, there are peculiar features of hydrogen and oxygen isotopes’ fractionation for carbohydrates, for example for cellulose. A correlation was shown between the values of water δ^18^O in leaves and stems [70], while the equilibrium of isotopic composition ^18^O/^16^O had specific temperature dependence, which changed little in temperatures of 20–30 °C (averaging 26‰) but increased to 31‰ at lower temperatures [71]. Either the existence of specific oxygen isotope composition in glucose derived from cellulose has been experimentally confirmed [72], when δ^18^O in the sample of glucose synthesized with the help of gluconeogenesis, differs from the sample obtained by photosynthesis, at least for the C-6 position. At the same time, it should be pointed out that the relatively low value of δ^18^O lactose, at least in part, is also a consequence of the aforementioned temperature sensitivity of isotopic fractionation [71] and is explained by the higher body temperature of an animal in comparison with the temperature of the plant leaves and stems.

### 2.2. Activating Influence of DDW at the Cell and Tissue Levels

The activating influence of DDW at the cell and tissue levels has been noted in a number of research works on the biological objects of animal and plant origin. For example, one of these research works showed the effect of ^2^H/^1^H for the life span of the unicellular biosensor *S. ambigua*, which had a parabolic pattern. In addition, the research observed the stimulating influence of DDW on the proliferative potential of cell culture (human dermal fibroblasts) in early passages [73]. Dynamics of the cell doubling index in the growth medium prepared on the base of DDW with δ^2^H = −807‰ showed a higher proliferation potential compared to water with normal isotopic composition: δ^2^H = −37‰ [5].

Another research shows the effect of liquid growth medium with δ^2^H = −904‰ for the speed of proliferation of human cultured adipose-derived stem cells which speed was characterized by cytotoxicity compared with the medium with the natural deuterium content (δ^2^H = −37‰). While that the presence of δ^2^H = −518‰ in medium led to a postponed (by one day) increase of migration and metabolic activity of adipose-derived stem cells [74,75]. Similar changes in cellular activity can also be explained by deuterium replacing with protium in НО–, НS– and H_3_N^+^–groups of macromolecules, especially in active and allosteric centers of enzymes, as well as by decrease of HDO (or ^1^HO^2^H) concentration in hydration shell of proteins and nucleic acids, which can change their thermodynamic and, therefore, thermokinetic parameters, stimulating metabolic and mitogenic processes in cells [76].

During study of influence of water с δ^2^H = −839‰ for rate of survival of DLD-1 cell line, it was found that DDW was able to stimulate mitochondrial activity and enhance apoptosis of DLD-1cells line [77]. Moreover, the miRNA pattern, which was isolated from exosomes, had significant differences in cells that were incubated in the liquid medium with δ^2^H = −839‰, in comparison with the medium with δ^2^H within the range from −76‰ till 0‰.

Experimental possibility of influence for activity of mitochondria isolated from rat liver using DDW with δ^2^H within the range from −705‰ till −665‰ and δ^18^О within the range from −135‰ till −11‰ has been confirmed in some research [78,79]. In that research it was found that in case of reduction of ^2^H and ^18^О content in the incubation medium the higher generation (by 35%) of hydrogen peroxide by mitochondria isolated from rats liver that consumed the water with low deuterium concentration was observed. The revealed change in functional activity of mitochondria indicates the ability of the animals’ organism to adapt to the deuterium-depleted drinking ration, that possibly is due to the formation of the transmembrane isotope gradient ^2^H/^1^H, which induces preadaptation of living system. The mechanism of the biological effect of the altered ratio of hydrogen stable isotopes can be explained by the direct relationship with the work cycle tricarboxylic acids. This is explained by the fact that the enzymes of the tricarboxylic acid cycle, localized in internal volume of mitochondria, provide for redistribution of deuterium between the cytoplasmic and mitochondrial water pools [56]. At the same time the reduction of δ^2^H in cytoplasmic water contributes, among other things, to normalization of cells phenotype with impaired metabolism regulation, for example, for cancer cell lines [80].

It is necessary to note the universal nature of deuterium low concentrations effect in cellular structures, as evidenced by change in metabolic and growth activity not only of animal cells, but also in cells of plant origin. For example, it was shown that incubation of corn seedlings (*Zeamays L.*) in DDW δ^2^H = −839‰ led to formation of airy cavities in the internal structure of a root [81]. In comparison with the control reference sample started in water δ^2^H = −69‰ it was found that deuterium-depleted water determines the best development of the absorbent bristles and increase in number of leading bundles and central vessels in the metaxylem. Also the change in ultrastructural and physiological characteristics of *Beta vulgaris* var. Conditiva seedlings was also shown under the term of hyperhydricity as a result of using the cultural medium with δ^2^H = −839‰ [82]. The structural changes (leaves and hypocotyls) characterized by modification of fluoroplastics, mixoplasma, tonoplast and nuclei, were related to hyperhydricity and were significantly less when *Beta vulgaris* var. Conditiva was grown in DDW.

### 2.3. Activating Influence of DDW for Organs and Organismic Level

More significant changes in comparison with molecular, organoid and cellular levels were observed during the study of DDW effects on internal organs and the organism as a whole. For example, the dependence of neuropsychiatric disorders (depressions) among the population on deuterium content in drinking water was researched. The correlation between the deuterium content in tap water and the level of depressions in US regions was researched. The increase of depressions frequency by 1.8% (*p* = 0.0016) along with increase of deuterium concentration in tap water for every δ^2^H = 64‰ was observed. The results were confirmed in experiments on laboratory animals, which on the background of simulated chronic stress, consumed water with δ^2^H = −411‰ (in control reference group animals consumed water with δ^2^H = −99‰). As a result, it was shown that the frequency of depressive-like signs was reduced in mice that consumed DDW [83], which is explained by its effect on the activity of serotonergic mechanisms of regulation of the nervous tissue functioning. The sensitivity of the central nervous system to fluctuations in the isotopic composition of water is confirmed by experiments on the Wistar rats, which consumed water with δ^2^H within the range from −827‰ to −807‰. They demonstrated, in comparison with the rats who drank water with δ^2^H within the range from −69‰ to −37‰, the reduction of fear and anxiety in unfamiliar environment [84]. In another research by the same authors the Wistar rats who consumed DDW showed an improvement in long-term memory and absence of short-term memory differences compared with animals that consumed water with a natural concentration of deuterium [85]. The decrease of HDO in the drinking ration also had a beneficial effect on prooxidant–antioxidant system of a brain during acute hypoxia in laboratory animals [86]. Studies have shown that in case of consuming DDW (δ^2^H = −665‰) for 8 weeks, there is a decrease of deuterium content in the blood plasma by 317‰ as well as in the brain of laboratory animals by 209‰, compared with the control group consuming natural water. Furthermore, DDW consumption in rat hypoxia modeling improves the functioning of antioxidant protection enzymes (catalase, superoxide dismutase, glutathione peroxidase and glutathione reductase) in the blood, increasing its antioxidant potential by 20%, while decreasing the intensity of free radical oxidation in plasma and the rate of peroxide modification of biomolecules in the red blood cells. Also in brain tissues of rats consuming DDW there was no disruption in catalase and superoxide dismutase functioning, and an increase (by 71%) in concentration of restored thiol-containing compounds, which reduce the risk of nerve cells damage under hypoxia. The presence of a neuroprotective effect is also confirmed by higher (by 32%) antioxidant activity of lyophilized brain tissue, as well as by a lower intensity of free radical oxidation (by 13%) and by the speed of oxidative modification of biomolecules (by 16%) in these tissues. The latter proves the feasibility of using the neuroprotective effects of DDW in case of cerebral circulation violations in experimental and clinical practice [87].

To influence nervous tissue, water with modified isotopic composition can affect the aging rate of the whole organism. For example, white outbred female rats of presenile age (20–22 months) consumed DDW (δ^2^H = −704‰) as drinking water for five weeks and it led to development of the expressed geroprotective effect, manifested in the appearance of recovery signs in the estrous cycle, as well as in the improvement of coat condition compared with the same parameters in animals exposed to drinking water with δ^2^H = −37‰. At the same time the rats that drank DDW showed development of persistent anti-stress adaptive responses of their bodies to calm and enhanced activation as well as increase in bactericidal activity of their skin. Thus in experiments on mammals the direct confirmation of DDW’s geroprotective properties was obtained, and the relation between geroprotective and anti-stress effects was shown when DDW was used in the drinking ration. The study revealed a more expressed decrease of deuterium content in the rats’ blood plasma (by 33.2%) in comparison with the change of deuterium content in visceral organs (liver—by 7.2%, kidney—by 9.7%, heart—by 7.3%). These changes led to formation of the experimental group of a new isotope ^2^H/^1^H gradient in the animals’ organisms, in which δ^2^H “visceral organs” > δ^2^H”blood plasma”, what is the opposite of physiological ^2^H/^1^H gradient, where δ^2^H “visceral organs” < δ^2^H”blood plasma” [88]. As the animals consumed food with unchanged isotopic composition under the experimental conditions, the observed changes are due only to the consumption of DDW. At the same time the decrease of deuterium content in blood plasma leads to decrease in its content in visceral organs also, obviously, due to replacement of deuterium with protium in hydroxyl (–ОН) and thiol (–SH) groups, as well as in primary and secondary amino groups (–NH_2_, =NH). The deuterium substitution by protium in active and allosteric centers of enzymes can alter the speed of catalytic reactions as a result of decrease in activation energy of transition states of the “enzyme-substrate” complex, which can serve as a basis for development of the organism’s metabolic adaptation and lead to occurrence of anti-stress reactions at the system level. In case of substitution of deuterium by protium, not only energy of covalent chemical bonds changes, but there are also differences in the intermolecular interaction forces (for example, due to change in hydrogen bonds energy) between some singular molecules [89]. The noted systemic changes, indicating the restoration of neuroendocrine regulation impaired in animals of pre-senile age, are apparently caused by a decrease of deuterium in blood and visceral organs (liver, kidney, heart), and also by the ease of DDW use as a nutritional factor, which allows us to consider it as a promising solution for holistic geriatric care in a presenile period of ontogenesis. The presence of isotopic gradient ^2^H/^1^H was also confirmed in the study of biological fluids in humans. So women who gave birth showed significant differences in natural conditions in δ^2^H of blood plasma (average δ^2^H = −74‰), oral fluid (average δ^2^H = 26‰) and breast milk (average δ^2^H = −91‰), which led to formation of isotopic gradient ^2^H/^1^H (δ^2^H oral fluid >> δ^2^H blood plasma > δ^2^H breast milk) [90]. These differences in the isotopic composition to a certain extent are caused by peculiarities of the biochemical composition of biological fluids; they are directly interrelated with the mass fraction of water (R*_Spearman_* = 0.81, *p* < 0.0001), and also negatively correlate with protein content (R*_Spearman_* = −0.55, *p* < 0.0005), carbohydrates (R*_Spearman_* = −0.80, *p* < 0.0001) and lipids (R*_Spearman_* = −0.82, *p* < 0.0001). This indicates the dependence of the deuterium concentration on the mass fraction of water and the content of carbohydrate and lipid substrates, which are characterized by approximately the same high rate R*_Spearman_*. This data can be explained by the lowest intensity of isotope exchange ^2^H/^1^H in hydrophobic (non-polar) radicals of lipids, which even under terms of water consumption with different isotopic composition make a stable contribution to the final concentration of deuterium in the cell. In addition, these relations between the content of biomolecules and deuterium must be taken into account while developing non-invasive methods for monitoring the content of heavy non-radioactive isotopes in a body, including deuterium, since the biochemical composition of biological fluids can be variable depending on the person’s lifestyle (diet, physical activity and other factors). It is not possible to explain completely the differences in content of ^2^H/^1^H isotopes only by characteristics of biochemical composition of these biological fluids and, apparently, there are additional mechanisms for regulation of isotopic metabolism in a body [91]. Among the possible mechanisms for isotopes fractionation, the presence of histohematic barriers shall be taken into account, including hematosalivar and hemato-lactation barriers, whose function is to ensure selective permeability for organic and inorganic molecules. Differences in intensity of metabolic processes in various tissues can make their contribution to difference in the isotopic composition in biological fluids [92], for example along with increased energy production there is an increased formation of water inside the cells from hydrogen isotopes which are part of biological oxidation substrates, i.e., different classes of organic compounds. At the same time, the metabolic water that formed intracellularly can significantly differ in deuterium content from extracellular water replenished in a body mainly as part of the diet [33]. It was also found that in case of consuming of DDW with δ^2^H = −615‰ by women it is possible to achieve a significant reduction in deuterium concentration in blood plasma (average δ^2^H = −175‰), oral fluid (average δ^2^H = −134‰) and also in breast milk but in less volume (average δ^2^H = −183‰), which leads to significant decrease of the described isotopic gradient ^2^H/^1^H (δ^2^H oral fluid > δ^2^H blood plasma ≥ δ^2^H breast milk) [90].

Changes in the direction and level of expression of isotopic gradients in a body can affect various periods of ontogenesis, including its morphofunctional parameters and activity of biochemical processes, providing short-term and long-term adaptation [78]. These body reactions can increase survival and life functions longevity [89]. The geroprotective influence of DDW was also confirmed on organisms with different levels of organization, including *Caenorhabditis elegans* (*C. elegans*), which were placed into the liquid medium with δ^2^H = −422‰ and δ^2^H = −229‰. It was found that in case of Mn-induced reduction of worms lifespan against the background of DAF-16 and SOD-3 inhibition, the use of DDW restored their expression and increased the lifespan of *C. elegans* [53].

The ability of water with a low content of heavy stable isotopes of hydrogen and oxygen ^18^О to neutralize the harmful environmental effects on a body has been shown in a number of research works devoted to study of not only chemical toxicants [93], but also to physical factors, for example, X-rays radiation [94]. The mice after exposure to γ-radiation ^60^Со at dose 1.0 Gray showed reduction of aging rate and reduction of frequency of lens opacities formation cases. In addition, the mature males of the *Balb/c* line which consumed water с δ^18^О= −206‰ and δ^2^H = −730‰ and were exposed to radiation ^60^Со at dose 0.50 Gray showed accelerated recovery of the thymus, spleen, and bone marrow in comparison with mice from the control reference group that consumed water with δ^18^О= −18‰ and δ^2^H = −103‰ [95]. After X-ray exposure, the mice which consumed water with δ^2^H = −807‰ and natural ^18^О content showed a protective effect on spleen structure accompanied by immune stimulation with an increase of megakaryocytes quantity [96]. In general, the mechanism of the protective effect of water with low content of HDO and ^1^H_2_^18^О is explained by its ability to stimulate the processes of proliferation, especially in radiosensitive tissues, which reduces damage to the body during sublethal irradiation [97]. While using of heavy water, radioprotective mechanisms is related to inhibition of catabolism and free radical oxidation of proteins, and also nucleic acids in radiosensitive organs and tissues.

It is planned to use the mechanisms of radioprotective action of water with depleted concentrations of heavy stable isotopes ^18^О and ^2^H described above during travel by humans in outer space [98,99].

### 2.4. Inhibitory Influence of DDW on Molecular and Organoid Level

Besides the activating effect of water with a modified isotopic composition, which is described in more researches, more often under physiological conditions and in the adaptation of the organism, the ability of DDW to inhibit certain metabolic reactions in the body is noted in a number of scientific works devoted to study of aspects of pathological metabolic states. Hence it is known that hydrogen ions are transported through the plasma membrane through the H^+^-ATPase (adenosin triphosphasatase), which cannot transfer deuterium atoms with the same ease as protium atoms [100]. Therefore, it is possible, that once the cell eliminates ions H^+^ to increase the pH by activating the Na^+^-H^+^ transport [101,102,103], the ^2^H/^1^H ratio is increased in the intercellular substance. So it is possible that the ^2^H/^1^H ratio will also regulate the cell cycle [104] if it reaches a certain threshold, thus triggering molecular mechanisms that transfer the cell into the S phase.

One of the possible mechanisms of DDW influence on the cell cycle can be carried out through the suppression of individual genes expression. Thus it was found that the water δ^2^H = −679‰ inhibited the cells proliferation at the G0/G1 stage and the S phase cell population [105]. Moreover, female mice which consumed water δ^2^H = −839‰ showed significant suppression of 7,12-dimethylbenz[*a*]anthracene (DMBA)-induced expression of Bcl2, Kras, and Myc [52]. In the other research the rats that consumed DDW (δ^2^H = −615‰) found a significant decrease in blood plasma of both the total amount of glycoproteins and their degree of glycosylation, which may reflect two processes: decrease of tumor-associated proteins expression, as well as decrease of healthy tissues detritus against the background of anticancer drugs use: Vinblastine, Cyclophosphamide, 5-Fluorouracil and Farmarubicine, due to introduction of DDW into the drinking ration [106].

Probably, DDW can selectively stress tumor cells [104], including its ability to regulate the activity of key genes in cell cycle without, however, interfering with metabolic processes in healthy tissues. This assumption is also confirmed by the fact that CBA/Ca-sensitive inbred mice’s consumption of drinking ration with δ^2^H = −865‰ resulted in (DMBA)-induced expression of c-myc, Ha-rasand p53 gene, which play a key role in the development of tumor cells. When evaluating the expression of RNA 48 h after exposure to a carcinogen, the expression of all the genes mentioned above was inhibited in various organs: spleen, lung, thymus, kidney, liver and lymph nodes [107].

### 2.5. The Inhibitory Influence of DDW at the Cellular and Tissue Levels

The inhibitory influence of DDW on cell proliferation has been established for cells with impaired metabolism, including in the neoplastic process. Thus it was shown that, in addition to expression of the gene regulating the cell cycle, DDW can change the activity of antioxidant defense enzymes, while inhibition of human breast cancer cell line (MCF7) was observed as the most expressed in the medium δ^2^H = −807‰. At the same time the activity of antioxidant protection enzymes (catalase, superoxide dismutase) and malondialdehyde content in MCF-7 cells changed. In general, the analysis of the cell cycle shows the ability of deuterium in low concentrations to cause a cell cycle stop during the G1/S transition [105]. Also changes in prooxidant-antioxidant system functional activity of nervous tissue cells against the background of their incubating in medium with δ^2^H = −679‰ led to decrease in mitochondrial potential of cerebellar neurons and their increased mortality rate during glucose deprivation and temperature stress (39 °C) [87].

Inhibition of cell growth with impaired metabolism was confirmed in research that studied 3-(4,5-dimethylthiazol-2-yl)-2,5-diphenyl tetrazolium bromide (MTT)-based cytotoxicity for cell lines: AGS (human gastric adenocarcinoma), MDA-MB231(human breast adenocarcinoma), U-87MG (glioblastoma multiform), PC-3 (human prostate adenocarcinoma) where DDW (within the δ^2^H range from −743‰ to −486‰) increased the inhibitory effect of paclitaxel on the cell lines of the mammary gland, prostate, stomach cancer and glioblastoma, and the effect was more expressed in cases of mammary gland and prostate [108].

In addition, the incubation medium δ^2^H = −807‰ significantly decreased the growth rate of fibroblast cell line. In the same research the inhibition of different human breast adenocarcinoma (MDA-MB-231 and MCF-7) cells growth in female CBA/Ca mice. The above described changes in cell lines growth rate can be caused by the effect of ^2^H/^1^H ratio, reduction of which results inter alia to the following:

—increasing of time required by the cell to achieve the threshold ratio of deuterium/protium, which ratio triggers cell division;

—neutralization of oncogenic activity of ATPase gene, which can behave as an oncogene in mammalian cells along with increased concentrations of deuterium in the medium. Therefore, the reduced removal of protons from the cell slow down their proliferation by reducing the deuterium/protium ratio [109];

—decreasing in proliferation, as even a small decrease in deuterium concentration in the intercellular substance is able to inhibit cell growth, because it does not allow the intracellular concentration of deuterium to raise up to the threshold level [61].

Moreover, DDW (δ^2^H = −679‰) influenced not only the proliferation, but also the migration of tumor cell lines (nasopharyngeal carcinoma and MC3T3-E1). At the same time activation of normal preosteoblast cell MC3T3-E1 growth was observed when it was cultured in DDW. The analysis of the cell cycle showed the ability of DDW to cause the cell cycle to stop during the G1/S transition, reducing the cells number in S-phase and contributing to increase of cells number in the G1 phase in tumor cells (nasopharyngeal carcinoma), also DDW suppressed the expression of the proliferating cell nuclear antigen matrix metalloproteinase 9 [80]. Similar results with changes in cell cycle phases were obtained in research on the human lung carcinoma cell line A549 and human embryonic lung fibroblasts HLF-1 with incubation in DDW (δ^2^H = −679‰). It was shown that reduction of ^2^H/^1^H ratio in medium led to DDW-induced cellular apoptosis [110].

### 2.6. Inhibitory Influence of DDW for Organs and Organism Level

The effects of water with modified isotopic composition on gene expression, the activity of enzymes of nonspecific defense, mitochondrial activity, cell proliferation and cell migration described above allow us understanding the individual systemic processes associated with DDW introduction into drinking diet. The inhibitory effect of deuterium concentration reducing in a body is mainly associated with decrease in tumor growth and metastasis rate, whereas healthy organs usually do not experience a negative effect when DDW is consumed by living organisms.

For example, in a clinical research that included 129 patients with small tumors (small cells and small cell lung cancers) who consumed DDW in addition to conventional chemotherapy and radiation therapy, an increase in median survival time was noted. That increase was 25.9 months in males and 74.1 months in female patients [52]. The other research demonstrated an increase in survival rate of patients with lung cancer and brain metastases who consumed the drinking ration with a final δ^2^H of −839‰ for at least 3 months. Also the decrease of metastatic spread in these patients was observed [111]_._ Also 91 patients with prostate cancer were examined. These patients consumed DDW with a progressively lowering deuterium content in water from −326‰ to −839‰. During this process the patients’ tumors volumes decreased as well as the level of prostate-specific antigen, which decreases in correlation with the consumption of DDW [112].

Moreover, while modeling tumors (H460 cells) by its injecting to *Balb/c* mice drinking DDW (δ^2^H = −679‰), within 60 days inhibition of the tumor was observed (approximately 30%) in comparison with the group that had the same model of tumor and did not receive DDW [110].

At the same time, some studies presented convincing data which showed expressed difference between therapy effect of the same nutritive substance in the cell line (in vitro) and in animals with orthotopic tumor. An interesting explanation of this matter was recently published in the article, which contemplated metabolic water as influenced factor to growth rate of cancer cells [113], that based on some sequential suggestions about metabolic pathways in brain tumors:

(1) glioma cells (in vivo) are metabolically flexible to use beta-hydroxybutyrate in catabolism (in contradict of RG2 and 9L glioma models [114]) as do healthy cells of the contralateral brain areas;

(2) under a natural condition ketogenic diet, which containing 91% fat and 9% protein [115], can increase production intracellular water about two times compared with carbohydrates, that occurs due to cooperate mitochondrial and peroxisomal lipid-substratum oxidation to H_2_O [116];

(3) in cells, between cytoplasmic and mitochondrial pools, the isotope (^2^H /^1^H) ratio change can take place, which leads to a decrease in deuterium content especially via oxidation long-chain unsaturated fatty acids in ^1^H_2_O in mitochondria with recycling matrix water by citric acid cycle’s enzymes [56];

(4) deuterium reduction in matrix water can regulate not only functional activity of mitochondria, but also speed of other cellular processes caused by transfer DDW to the cytosol, and as result of the foregoing, cell ATP-production [78,79] and interfacial protein interactions are changed and, consequently, cellular rate of growth can be various due to deuterium loaded in molecular structures of living cell.

Nevertheless, the using of ketogenic diet in tumor therapy can be accompanied by a decrease in vivo in the intensity of fatty acid β-oxidation. It is possible, because speed of short-chain fatty acids (C_6_-C_10_) β-oxidation in mitochondria is higher than cooperative “peroxisomal-mitochondrial” long-chain and unsaturated fatty acid oxidation [117]. Therefore, during long-lasting lipid loading, it is can be probably to drop down ATP-production especially under intracellular O_2_ fault (for example, produced by circulatory disorders of tumor), acetyl-CoA deficiency forming, accumulate ketogenic substrates (β-hidroxibutiryl-CoA and acetoacetyl-CoA), decrease pH and other processes, but deuterium depletion does not occur in the cytosol water. In contrast of mitochondrial ^1^H_2_O-production, the DDW consumption more significantly changes deuterium content in cytosol and less dependent on non-physiological cell conditions, so it can be used more effectively in adjuvant therapy.

## 3. Conclusions

This review demonstrated the important role of water isotopic composition to ensure the passing of many biochemical reactions, regulation of energy metabolism and functional activity of mitochondria, changes in the speed of the cell cycle, increase the body’s adaptation and stimulate a number of vital processes in healthy tissues. At the same time some of the biological effects of DDW described above (antioxidant, antidepressant, anticancer, hypoglycemic, etc.) can be used in therapy of various diseases in humans. The stimulating effect on living systems of different levels of organization (molecular, organoid, cellular, tissue, organ and organism levels) is mainly produced by water with δ^2^H within the range from −229‰ to −679‰ and δ^18^O within the range from −135‰ to −206‰.

Nevertheless, it shall be noted that the consumption of water with a modified isotopic composition which has a significant deviation from the natural concentrations of ^2^H and ^18^O isotopes is also accompanied by stress effect on a body. This can be caused, for example, by a sharp change of isotope ^2^H/^1^H gradient from “δ^2^H_blood plasma_ > δ^2^H_visceral organs_” to “δ^2^H_blood plasma_ << δ^2^H_visceral organs_”, therefore, the consumption of the drinking ration with δ^2^H below −679‰ by living organisms may lead to isotopic shock [118], which has, inter alia, an inhibitory effect on biological processes and inhibits in some cases the growth, division and migration of cells, including cells of tumoral origin. The presence of an isotopic gradient in human biological fluids is confirmed by differences in the content of deuterium in the series: δ^2^H_saliva_ >> δ^2^H_blood plasma_ > δ^2^H_breast milk_ (^2^H/^1^H gradient was equal to 117‰). At the same time while drinking deuterium-depleted water it is possible to achieve a significant reduction in the described isotope ^2^H/^1^H gradient by 58%: δ^2^H_saliva_ > δ^2^H_blood plasma_ ≥ δ^2^H_breast milk_ (^2^H/^1^H gradient was equal to 49‰). In addition to the drinking diet, the proportion of proteins, lipids and carbohydrates in the consumed food, which proportion is associated with oxidation of organic compounds in mitochondria. At the same time, the greatest decrease of deuterium content in intracellular water is achieved with increase of lipid-containing nutrients proportion in the diet. So, cooperative influence of the drinking/ingested DDW and diet (containing lipogenic sources with certain deuterium/protium ratio [113], which is naturally lower than it takes place in the food carbohydrates and proteins [90,91]) can be used for treatment based on the compartmentalized deuterium disequilibrium and changing of intracellular isotope ^2^H/^1^H gradient, especially in previously drug-treated cells [119]. Besides, the results of the studies make it possible to point to the ability of DDW to increase the potential of the protective systems of the human body, mainly with its preventive consumption at the stage of preparing the living system for the expected stress. Therefore, in clinical practice, DDW usage in the acute period of the development of pathology requires caution in connection with the risk of a synergistic negative effect on the body’s defense systems (both from the DDW side and from the pathological process [76,93]). Based on the foregoing, it is possible to recommend that people consume DDW during a period of remission of some chronic diseases, or when preparing the organism for extreme impacts (for example, high sports activities), and using it as a geroprotective nutritional factor of holistic geriatric care in presenile period [89]. The DDW also can be used as synergistic anti-inflammatory agent against sepsis with modulated oxidative stress/antioxidant parameters [120], as adjuvant to conventional anticancer treatment [52,105,111], moreover, DDW drinking is effective against hypoxia of the central nervous system and for the prevention of individuals with depression [83,84,85,86,87].

Also the influence of DDW for nucleic acids is noted, including its ability to increase the intensity of mitochondrial activity and autophagy as well as to alter and miRNA transcriptomic pattern in cells of some tumors [77,121]. Due to this, DDW can be used as adjuvant therapy for cancer treatment.

In its turn the influence of DDW on the molecular dynamics of DNA can be explained by several mechanisms. It is known, that decrease of deuterium concentration in functionally active regions of a DNA molecule may increase the rate of transcription and replication [98]. The latter effect can be caused by the influence of deuterium on the probability of occurrence of open states between complementary pairs of nitrogenous bases, which can significantly change the speed of reading genetic information. In the physiological range, the deuterium atom increases the probability for rupture the bond between the complementary nitrogenous bases by 0.22%−0.60%, which reflects its ability to slow down the reading speed of genetic information in transcription processes, narrowing down the range of regulatory mechanisms for persistent exposure during the cell cycle of the low-intensity adverse factor and leading to a decrease in the adaptive capacity of the cell [65].

At the same time in the case of occurrence of conditions weakening the strength of hydrogen bonds between bases in DNA molecule, the presence of a deuterium atom increases the rate of occurrence of open states, thus increasing the risk of mutations due to the greater availability of nitrogenous bases to the damaging effects of adverse external factors. The latter effect confirms the possibility of increasing the frequency of spontaneous mutations mediated by influence of deuterium atoms on the molecular dynamics of double-stranded DNA, which can play a significant role in the evolution of living organisms. All this is stipulated by the disparity of thermodynamic/kinetic effects associated with substitution of deuterium with protium in the DNA molecule, which indicates the ability of ^2^H/^1^H exchange to regulate the speed of vital processes of biologically active systems (for example, in reading of genetic information). Thus, the probability that living organisms have special mechanisms at different levels of organization that make long-term adaptation to pronounced fluctuations in ^2^H/^1^H ratio in the environment is not excluded [66]. Therefore even if only one protium atom is substituted by deuterium in the DNA molecule and at the same average DNA replication rate, some individual periodic decelerations can occur and accelerations equivalent in this case to the total intensity, although in general they are level with each other, but they are able to change the in-line reading pattern of genetic information which leads to a general accumulation of errors in the reproduction of genetic information, accompanied by a transition of quantitative changes (a number of replication failures) into qualitative defects (mutations) leading to persistent disorders of the genome in living beings, including inherited disorders.

Besides, when DDW is consumed, there is a decrease of HDO molecule content in the hydration shell of nucleic acids and proteins, which is also accompanied by changes in the biological activity of these macromolecules, for example, due to a higher frequency and amplitude of oscillations of atomic groups formed only from light isotopes. These rearrangements of biochemical processes in tissues with high metabolic activity are able to affect the morphofunctional parameters and long-term adaptation of the whole organism.

The considered biological effects that occur when the deuterium content in the organism decreases are important not only for slowing down aging and correcting biochemical disorders in humans in their natural environment, but also offer a perspective in the near future to use DDW during flights in outer space. In addition, it is known that water ice in the polar reservoirs of Mars is enriched in deuterium to at least 7000‰ [122], therefore, in the future it is necessary to study the possibility of correcting the negative effects of such water on living systems with DDW.
